# Relationship between increased systemic immune-inflammation index and coronary slow flow phenomenon

**DOI:** 10.1186/s12872-022-02798-0

**Published:** 2022-08-08

**Authors:** Xin-tong Dai, Tian-zhong Kong, Xiao-jiao Zhang, Bo Luan, Yong Wang, Ai-jie Hou

**Affiliations:** Department of Cardiology, The People’ S Hospital of China Medical University, The People’ S Hospital of Liaoning Province, No. 33, Wenyi Road, Shenhe District, Shenyang City, Liaoning Province, China

**Keywords:** Systemic immune-inflammation index, TIMI frame count, Coronary slow flow phenomenon, Coronary angiography, Inflammation

## Abstract

**Background:**

Systemic immune-inflammation index (SII, platelet × neutrophil/lymphocyte ratio), a new marker of inflammation, is associated with adverse cardiovascular events, but its relationship with coronary slow flow phenomenon (CSFP) is unclear. Therefore, we aimed to investigate the relationship between SII and CSFP.

**Methods:**

We enrolled consecutive patients who presented with chest pain, with normal/near-normal coronary angiography findings (n = 89 as CSFP group; n = 167 as control group). The baseline characteristics, laboratory parameters and angiographic characteristics of the two groups were compared.

**Results:**

SII levels were significantly higher in the CSFP group than in the control group (409.7 ± 17.7 vs. 396.7 ± 12.7, *p* < 0.001). A significant positive correlation between SII and the mean thrombolysis in myocardial infarction frame count (mTFC) was found (r = 0.624, *p* < 0.001). SII increased with the number of coronary arteries involved in CSFP. In multivariate logistic regression analysis, SII/10 was an independent predictor of CSFP (odds ratio: 1.739, *p* < 0.001). In addition, the SII level > 404.29 was a predictor of CSFP with 67.4% sensitivity and 71.9% specificity.

**Conclusions:**

SII can predict the occurrence of CSFP.

## Background

Coronary slow flow phenomenon (CSFP) is characterized by normal/near-normal epicardial coronary arteries (stenosis < 40%) but delayed vessel opacification in the coronary angiogram (CAG) [1, 2]. The prevalence of CSFP in patients undergoing CAG for suspected coronary artery disease (CAD) ranges from 1 to 7% [3].Although studies have shown that inflammation, oxidative stress, diffuse atherosclerosis, microvascular vasomotor and endothelial dysfunction are associated with CSFP [4–8], the exact pathogenesis of CSFP is unknown.

On the other hand, systemic immune-inflammation index (SII) is a novel marker of inflammation and is related to cardiovascular disease involving mechanisms such as atherosclerosis and inflammation [9–12], but the relationship with CSFP is unclear. Therefore, in view of the pathogenesis of CSFP and the significance of SII in cardiovascular disease, the aim of this study was to evaluate the relationship between increased SII and CSFP.

## Methods

### Study population

**Fig. 1:**
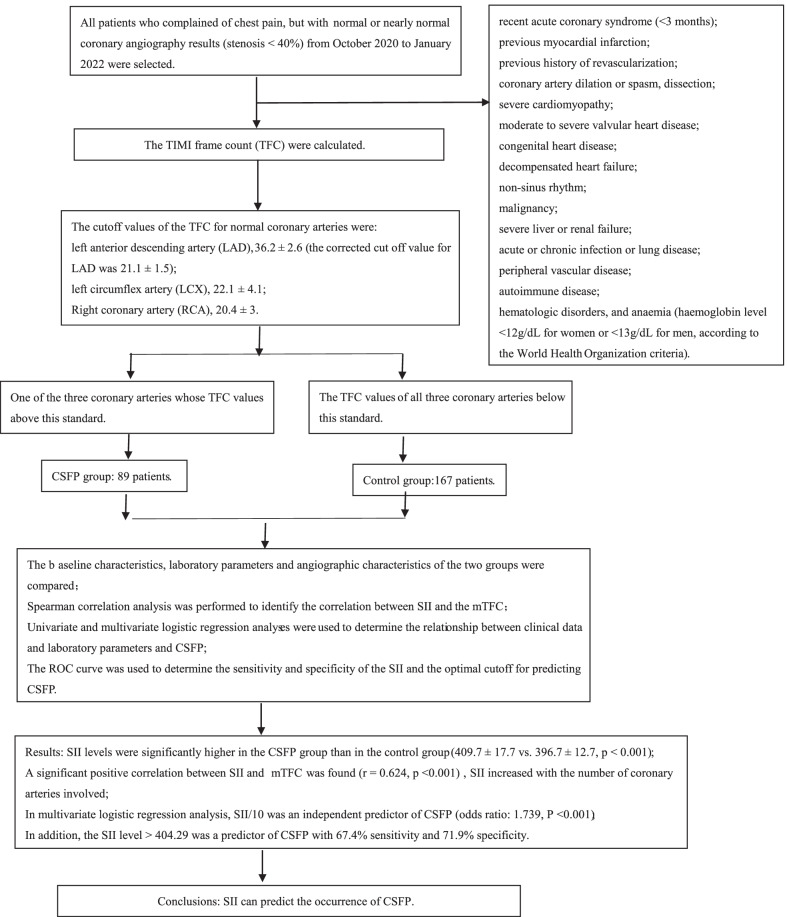
Study flow diagram

All patients who complained of chest pain, but with normal or nearly normal coronary angiography results (stenosis < 40%) from October 2020 to January 2022 at the People's Hospital of Liaoning Province were selected. Baseline clinical data, laboratory and angiographic data of all patients were retrospectively analysed. [13]. Patients with one of the three coronary arteries whose TFC values above this standard were considered to have CSFP while those with TFC values of all three coronary arteries below this criterion were considered controls [14]. Finally, 89 patients with CSFP and 167 patients with normal coronary blood flow were included in this study (Fig. 1). The exclusion criteria were as follows: recent acute coronary syndrome (< 3 months), previous myocardial infarction, previous history of revascularization, coronary artery dilation or spasm, dissection, severe cardiomyopathy, moderate to severe valvular heart disease, congenital heart disease, decompensated heart failure, non-sinus rhythm, malignancy, severe liver or renal failure, acute or chronic infection or lung disease, peripheral vascular disease, autoimmune disease, hematologic disorders, and anaemia (haemoglobin level < 12 g/dL for women or < 13 g/dL for men, according to the World Health Organization criteria) [15]. The study protocol was approved by the Institutional Ethics Committee and informed consent was obtained from all individuals involved in the study. This study was conducted in accordance with the principles of the Declaration of Helsinki.

### Coronary angiography

All patients underwent coronary angiography via radial or femoral approach using the Judkins technique for typical chest pain. The coronary arteries were observed at 30 frames/second (fps). Two experienced cardiologists measured coronary blood flow velocity using the TFC, while blinded to the patients' clinical parameters [13]. The TFC was calculated based on the difference between the first and last frames, the initial frame referred to the contrast agent filling more than 70% of the arterial lumen in a prograde direction, while the last frame showing the contrast agent appearing at distal coronary landmarks, which were defined as follows: the distal landmark of the LAD was the distal bifurcation (i.e., the "moustache"), usually at the apex of the heart; that of the LCX was the distal bifurcation of the longest branch, and in the RCA, it was the first side branch of the posterolateral artery. As the LAD artery is usually longer than the other major coronary arteries, the TFC of LAD was divided by 1.7 to obtain the corrected TFC. The cut off values for normal epicardial coronary arteries filling were 36.2 ± 2.6 frames for the LAD (21.1 ± 1.5 frames for the corrected cutoff value for LAD), 22.2 ± 4.1 frames for the LCX, and 20.4 ± 3 frames for the RCA [13]. Patients with one of the three coronary arteries whose values above this threshold were defined as CSFP. The mean thrombolysis in myocardial infarction frame count (mTFC) was defined as the sum of the TFC of the LAD, LCX and RCA divided by 3 [13].

### Laboratory measurements

The baseline characteristics of all patients were reviewed and blood samples were collected from the cubital vein in the forearm after 12 h of fasting prior to the coronary angiography. Laboratory parameters (including complete blood count, biochemical parameters and lipid parameters collected before coronary angiography) were recorded for all patients. And the neutrophil to lymphocyte ratio (NLR), platelet to lymphocyte ratio (PLR) were calculated. Total preoperative peripheral platelets count (P) × NLR were used to obtain SII (SII = P × N/L) [9].

### Statistical analysis

Statistical analysis was performed using SPSS software version 26.0. Continuous variables were presented as mean ± standard deviation, and categorical variables were presented as numbers and percentages. The differences between groups were compared using Student t test for continuous variables and the Chi-square (χ2) test was used for comparison of categorical variables. Spearman correlation analysis was used to identify the correlation between SII and mTFC. Univariate and multivariate logistic regression analyses were performed to determine the relationship between clinical data and laboratory parameters and CSFP. The factors showing statistical significance at the *p* < 0.05 level between groups were included in the univariate regression model, and those with *p* < 0.05 in the univariate regression analysis were finally analyzed by multivariate logistic regression. The receiver operating characteristic (ROC) curve was used to determine the sensitivity and specificity of the SII and the optimal cut off for predicting CSFP. A P value of < 0.05 was considered statistically significant.

## Results

**Fig. 2:**
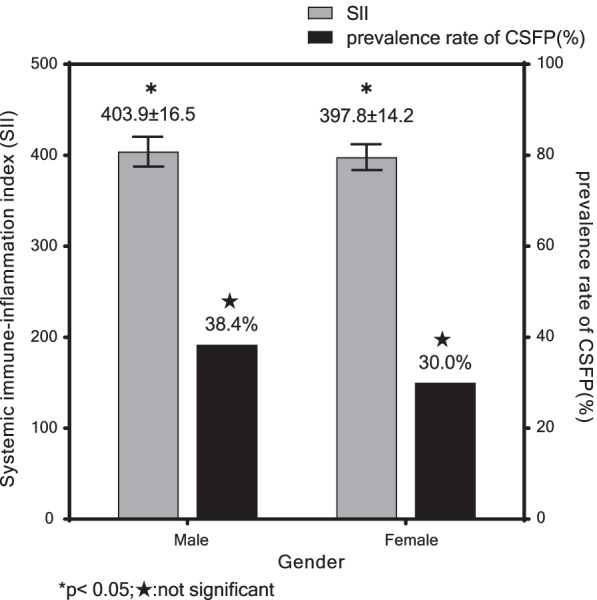
All patients were divided into two groups by gender to compare the SII and the prevalence of CSFP between male and female

**Fig. 3:**
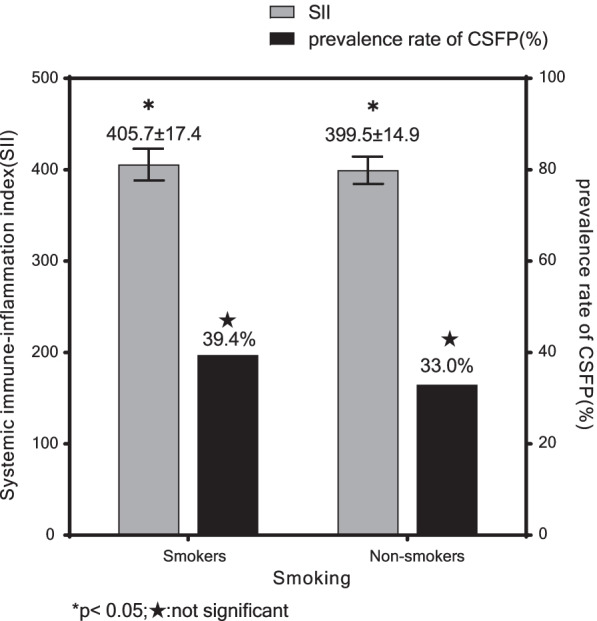
All patients were divided into two groups by whether smoking or not to compare the SII and the prevalence of CSFP between smokers and non-smokers

A total of 256 patients (89 in the CSFP group and 167 in the control group) were enrolled in this study. Baseline characteristics of the two groups are demonstrated in Table 1. There were no statistically significant intergroup differences in terms of age, gender, BMI, and smoking (p > 0.05), but the incidence rates of hypertension (36.0% vs. 24.0%, p = 0.042) and diabetes mellitus (29.2% vs. 18.0%, P = 0.038) in the CSFP group were significantly higher than those in the control group. No statistically significant differences were observed in medication between the two groups (P > 0.05). In this study, patients were also divided into two groups respectively according to gender and whether smoking or not to compare the incidence of CSFP and SII (Figs. 2, 3). The results showed that SII was significantly higher in male (403.9 ± 16.5 vs. 397.8 ± 14.2, p = 0.002); and smokers (405.7 ± 17.4 vs. 399.5 ± 14.9, p = 0.005). Although there were no significant differences in the incidence of CSFP in the gender and smoking group, the prevalence of CSFP was also higher in male and smokers in the study.

**Table 1:** Baseline characteristics of the two groups

	CSFP group (n = 89)	Control group (n = 167)	*p* value
Age, years	59.6 ± 5.6	58.5 ± 6.3	0.193
Male sex, n (%)	56 (62.9)	90 (53.9)	0.165
BMI, kg/m^2^	25.4 ± 1.3	25.7 ± 2.1	0.160
Smoking, n (%)	28 (31.5)	43 (25.7)	0.331
Hypertension, n (%)	32 (36.0)	40 (24.0)	0.042
Diabetes mellitus, n (%)	26 (29.2)	30 (18.0)	0.038
Calcium canal blocker, n (%)	16 (18.0)	27 (16.2)	0.712
Beta-blocker, n (%)	20 (22.5)	32 (19.2)	0.531
ACEI/ARB, n (%)	13 (14.6)	29 (17.4)	0.570
Antiplatelet, n (%)	22 (24.7)	45 (26.9)	0.699
Statin, n (%)	20 (22.5)	39 (23.4)	0.873
Nitrates, n (%)	12 (13.5)	26 (15.6)	0.655

Values are mean ± standard deviation or numbers with percentages in parentheses

CSFP: Coronary slow flow phenomenon, ACEI: angiotensin-converting enzyme inhi-

bitor, ARB: angiotensin II receptor blocker

**Fig. 4:**
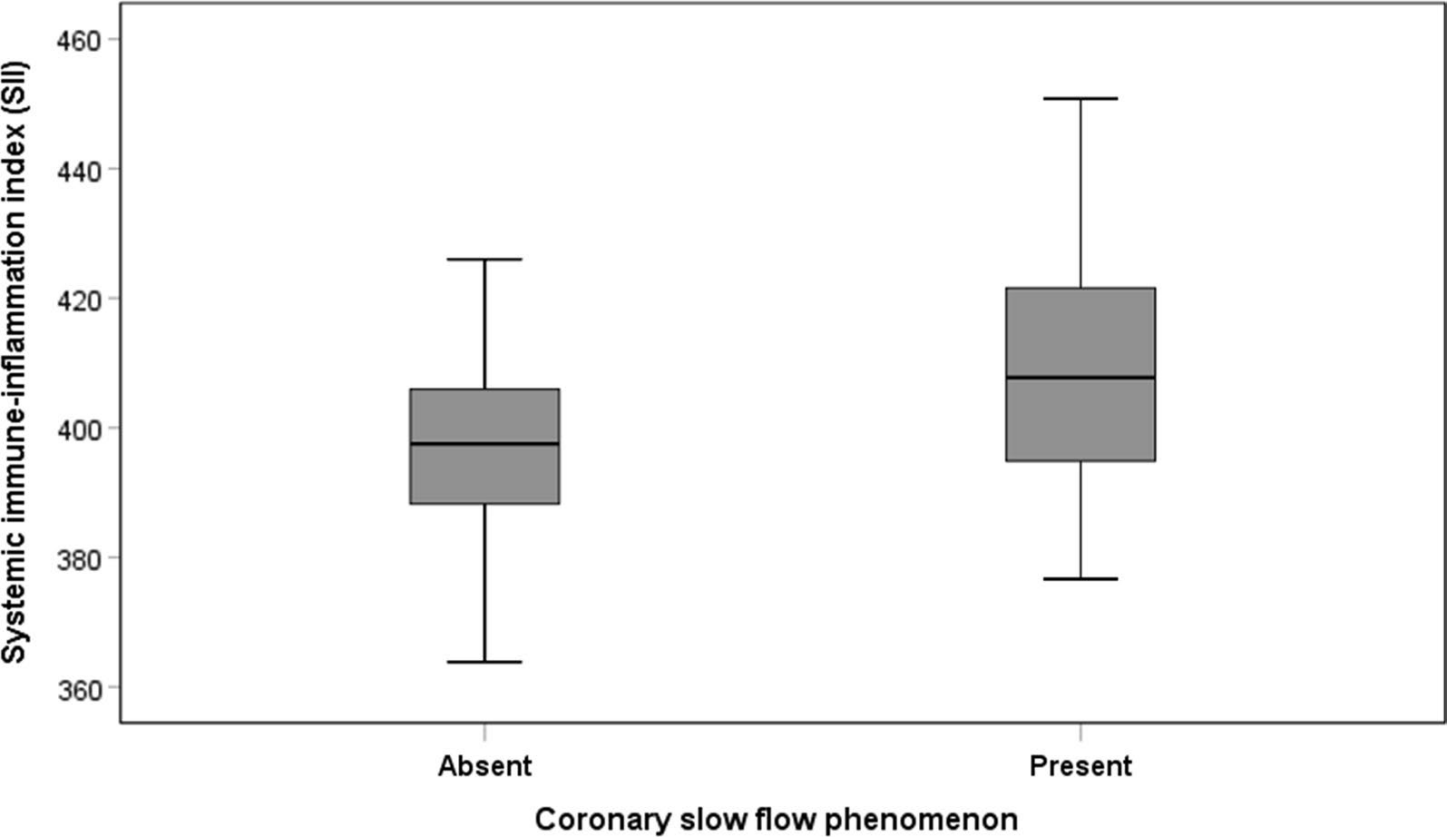
Systemic immune-inflammation index (SII) according to the presence or absence of coronary slow flow phenomenon

**Fig. 5:**
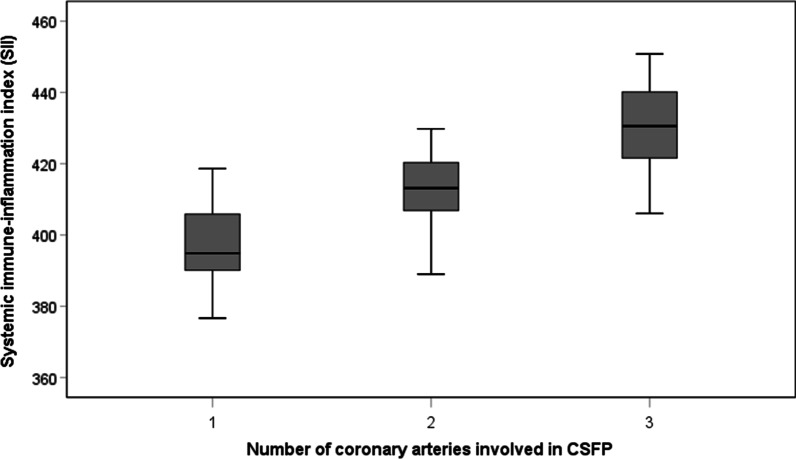
Correlation between the number of coronary arteries involved in CSFP and systemic immune-inflammation index (SII)

The laboratory parameters of the two groups are shown in Table 2. There were no statistically significant differences between the two groups in the following laboratory values: neutrophil count, lymphocyte count, monocyte count and eosinophil count, haemoglobin and total cholesterol, triglyceride, low-density lipoprotein (LDL) cholesterol and urea, creatinine, uric acid, mean platelet volume, and PLR (P > 0.05). The white blood cell count (6.8 ± 1.2 vs. 6.4 ± 1.0*10^3^/mm^3^, p = 0.006), platelet count (235.7 ± 9.7 vs. 232.5 ± 13.2*10^3^/mm^3^, p = 0.031) in the CSFP group as well as fasting plasma glucose (5.1 ± 0.5 vs. 5.0 ± 0.6 mmol/l, p = 0.034), NLR (1.74 ± 0.09 vs. 1.71 ± 0.12, p = 0.034) and SII (409.7 ± 17.7 vs. 396.7 ± 12.7, *p* < 0.001, Fig. 4) were significantly higher than in the control group, and SII increased with the number of vessels in which CSFP occurred (Fig. 5). However, high-density lipoprotein (HDL) cholesterol (1.0 ± 0.1 vs. 1.1 ± 0.2 mmol/L, P = 0.030) was significantly lower than in the control group.

**Table 2:** Laboratory parameters of the two groups

	CSFP group (n = 89)	Control group (n = 167)	*p* value
White blood cell, 10^3^/mm^3^	6.8 ± 1.2	6.4 ± 1.0	0.006
Neutrophils, 10^3^/mm^3^	3.50 ± 0.32	3.46 ± 0.45	0.407
Lymphocytes, 10^3^/mm^3^	2.01 ± 0.16	2.03 ± 0.28	0.543
Monocyte, 10^3^/mm^3^	0.5 ± 0.1	0.4 ± 0.1	0.105
Eosinophils, 10^3^/mm^3^	0.2 ± 0.1	0.1 ± 0.1	0.135
Haemoglobin, g/l	146.7 ± 9.7	145.8 ± 9.1	0.503
Platelets, 10^3^/mm^3^	235.7 ± 9.7	232.5 ± 13.2	0.031
Total cholesterol, mmol/l	4.6 ± 0.4	4.7 ± 0.5	0.120
Triglyceride, mmol/l	1.5 ± 0.2	1.4 ± 0.3	0.112
HDL-C, mmol/l	1.0 ± 0.1	1.1 ± 0.2	0.030
LDL-C, mmol/l	3.0 ± 0.2	2.9 ± 0.3	0.136
Fasting plasma glucose, mmol/l	5.1 ± 0.5	5.0 ± 0.6	0.034
Urea, mmol/l	5.3 ± 0.7	5.1 ± 0.6	0.121
Creatinine, umol/l	73.2 ± 9.3	71.3 ± 9.9	0.125
Uric acid, umol/l	322.1 ± 27.3	313.7 ± 52.1	0.091
Mean platelet volume, fl	10.3 ± 1.0	10.0 ± 1.0	0.105
Neutrophil to lymphocyte ratio	1.74 ± 0.09	1.71 ± 0.12	0.034
Platelet to lymphocyte ratio	117.7 ± 10.1	116.6 ± 17.2	0.513
SII	409.7 ± 17.7	396.7 ± 12.7	< 0.001

CSFP: Coronary slow flow phenomenon, HDL-C: high-density lipoprotein choleste-

rol, LDL-C: low-density lipoprotein cholesterol, SII: systemic immune-inflammation

index

The angiographic characteristics of patients in the two groups are shown in Table 3. In the CSFP group, the corrected TFC for LAD (43.2 ± 24.8 vs. 19.7 ± 1.0, *p* < 0.001), and the TFC for LCX (29.8 ± 10.1 vs. 18.8 ± 2.2, *p* < 0.001), for RCA (27.9 ± 8.8 vs. 17.2 ± 2.2, *p* < 0.001) and the mean TFC (33.6 ± 9.4 vs. 18.6 ± 1.6, *p* < 0.001) were significantly higher than those in the control group. In the CSFP group, a total of 47 (52.8%) patients developed CSFP in the LAD, 54 (60.7%) patients showed CSFP in the LCX, and 61 (68.5%) in the RCA. Moreover, 37 (41.6%) patients had CSFP in one major coronary artery, 31 (34.8%) in two major coronary arteries, and 21 (23.6%) in three coronary arteries. The mean number of coronary arteries with CSFP was 1.82 ± 0.79.

**Table 3:** Angiographic characteristics of the two groups

	CSFP group (n = 89)	Control group (n = 167)	*p* value
TIMI frame count			< 0.001
LAD (corrected)	43.2 ± 24.8	19.7 ± 1.0	
LCX	29.8 ± 10.1	18.8 ± 2.2	
RCA	27.9 ± 8.8	17.2 ± 2.2	
mean TFC	33.6 ± 9.4	18.6 ± 1.6	
Distribution of CSFP among major coronary arteries			
LAD, n (%)	47 (52.8)		
LCX, n (%)	54 (60.7)		
RCA, n (%)	61 (68.5)		
Number of coronary arteries involved			
1, n (%)	37 (41.6)		
2, n (%)	31 (34.8)		
3, n (%)	21 (23.6)		
Average number of coronary arteries with CSFP	1.82 ± 0.79		

CSFP: Coronary slow flow phenomenon, TIMI: thrombolysis in myocardial infarction, LAD: left anterior descending artery, LCX: left circumflex artery, RCA: right coronary artery, TFC: TIMI frame count

**Fig. 6:**
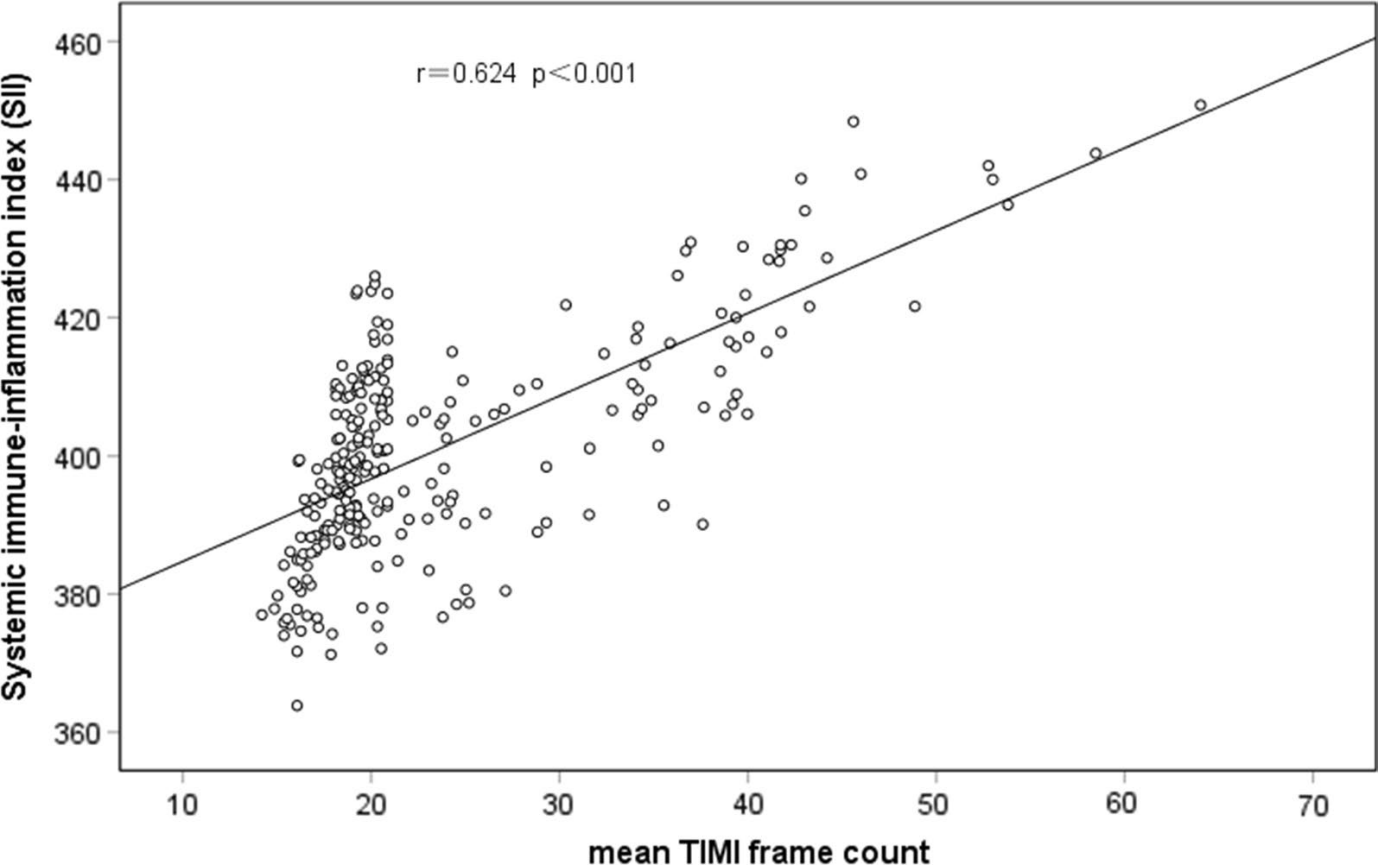
Correlation analysis between systemic immune-inflammation index (SII) and the mean TFC

In the correlation analysis, a significant positive correlation was found between SII and mTFC (r = 0.624, *p* < 0.001; Fig. 6). Multivariate logistic regression analysis revealed that SII/10 was an independent predictor of CSFP (odds ratio: 1.739, 95% confidence interval [CI] 1.408–2.148, *p* < 0.001), i.e., each 10-unit increase in SII was associated with a 73.9% increase in CSFP prevalence in the study population (Table 4).

**Table 4:** Univariate and multivariate logistic regression analysis for presence of CSFP

Variables	Univariate regression analysis		Multivariate regression analysis	
	Odds ratio (95% CI)	*p* Value	Odds ratio (95% CI)	*p* Value
Hypertension	1.782 (1.018–3.121)	0.043	1.342 (0.678–2.654)	0.399
Diabetes mellitus	1.885 (1.030–3.448)	0.040	1.070 (0.503–2.277)	0.860
White blood cell	1.420 (1.099–1.833)	0.007	1.146 (0.863–1.523)	0.347
Platelet	1.022 (1.000–1.045)	0.051		
HDL-C	0.214 (0.047–0.987)	0.048	0.243 (0.045–1.321)	0.102
Fasting plasma glucose	1.645 (1.032–2.621)	0.036	1.661 (0.992–2.781)	0.054
Neutrophil to lymphocyte ratio	10.080 (0.994–102.209)	0.051		
SII/10	1.804 (1.474–2.207)	< 0.001	1.739 (1.408–2.148)	< 0.001

CSFP: Coronary slow flow phenomenon, HDL-C: high-density lipoprotein cholesterol, SII: systemic immune-inflammation index

**Fig. 7:**
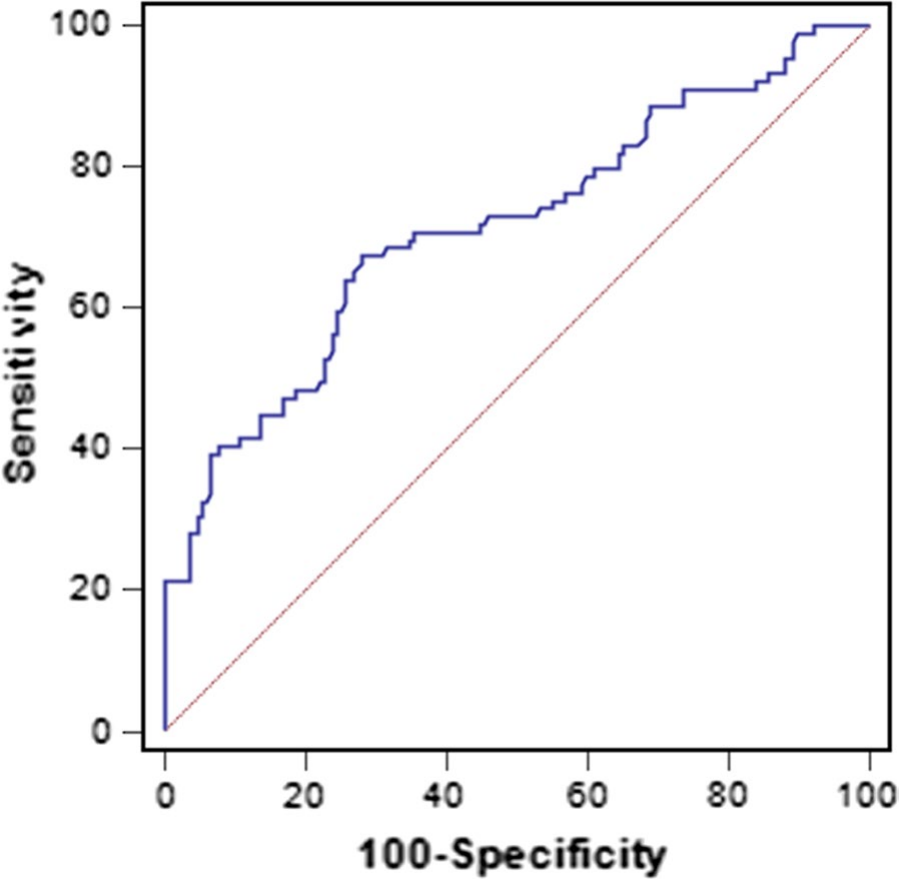
Receiver operating characteristic (ROC) curve analysis for systemic immune-inflammation index (SII) for predicting CSFP

The ROC curve was used to evaluate the discriminatory capability of SII for the occurrence of CSFP. The ROC curve showed an area under the curve of 0.715 (95% CI 0.655–0.769, *p* < 0.001; Fig. 7), and using a cut off level of 404.29, SII predicted the presence of CSFP with an 67.4% sensitivity and 71.9% specificity.

## Discussion

In this study, we found that higher SII before coronary angiography was significantly and independently associated with the presence of CSFP. The SII level in patients with CSFP was significantly elevated and increased with the number of vessels involved and was also positively correlated with mTFC. Multivariate regression analysis indicated that SII/10 was an independent predictor of CSFP. To the best of our knowledge, this is the first study in the literature reporting the relationship between SII and CSFP.

CSFP is an angiographic phenomenon with specific pathogenesis and diagnostic criteria [2], which usually occurs in young men, smokers and those with comorbid metabolic syndrome [16]. Similarly, in our study, CSFP was more common in male, smokers, and patients with diabetes mellitus and hypertension. The main clinical symptom of CSFP is unstable angina pectoris, although CSFP is usually a benign phenomenon, it has also been reported to be associated with life-threatening adverse cardiovascular events such as acute coronary syndrome, ventricular fibrillation, and sudden cardiac death [17]. The exact pathophysiological mechanisms of CSFP are still unclear, but inflammation [4, 18], diffuse atherosclerosis [8, 19], microvascular [20] and endothelial dysfunction [7], and oxidative stress [21] have been thought to be involved. In addition, cardiovascular risk factors such as diabetes mellitus and hypertension [22, 23], lipid index such as HDL cholesterol and triglyceride, and conventional clinical parameters including fasting glucose, uric acid, etc. were also considered to be associated with CSFP [16, 24, 25]. As in line with previous studies, we also found the prevalence of diabetes mellitus and hypertension and fasting glucose levels were significantly higher in the CSFP group, whereas HDL cholesterol was significantly lower in the CSFP group as compared with the control group.

Inflammation plays an important role in the development of CSFP, neutrophils can infiltrate endothelial tissue and release pro-oxidants and pro-inflammatory mediators, which in turn can form neutrophil extracellular traps (NETs) and promote the formation and development of atherosclerotic plaques [26, 27]. Doğan et al. [15] reported NLR as an inflammatory marker to be associated with the presence of CSFP. It has also been reported that high sensitive CRP (hs-CPR) may be an early indicator that could predict the occurrence of CSFP [18]. While SII as an inflammatory marker has been considered to predict the occurrence of some cardiovascular diseases. for example, one study showed that SII can act as a circulating immune inflammatory cell to predict major cardiovascular events after coronary intervention in patients with coronary heart disease [9]. Another study concluded that elevated SII may have a predictive value for coronary artery dilation [28]. Meanwhile, CSFP as a common cardiovascular disease, this study also confirmed that SII can be used as a predictor of CSFP. And the results show that the values of white blood cell count, NLR, and SII were all significantly higher in the CSFP group than in the control group. On the other hand, lymphocyte levels decrease in number during chronic inflammation due to stress response. In addition to increased apoptosis, downregulation of proliferation and redistribution of lymphocytes can lead to low lymphocyte counts [29, 30]. Furthermore, a decrease in lymphocyte counts also has an effect on cardiovascular disease, as found by Major et al. [31] in experimental studies in B-cell-deficient mice where a low lymphocyte count promoted atherosclerosis, and another study also showed that low lymphocyte counts were associated with poor prognosis in cardiovascular disease [32]. Similarly, our study showed that patients in the case group had lower lymphocyte counts and higher NLR, PLR and SII. These findings all suggest that SII, an indicator of inflammation, may be a causative factor for CSFP. Platelets play an important connecting role in inflammation, thrombosis, and atherosclerosis formation. Platelets can recruit leukocytes and monocytes to the site of inflammation and secrete inflammatory mediators such as chemokines and cytokines, which can lead to vascular inflammation [33]. At the same time, enhanced thrombosis is related to the development of CSFP as well, and it has been documented that the platelet activation ability is enhanced in CSFP patients, when compared with controls [34]. Akboga et al. [22] also found that PLR was not only significantly correlated with CSFP as an inflammatory indicator, but also could lead to the occurrence of CSFP through enhanced pro-systemic coagulant activity. The results of this study also confirmed that platelet, PLR, and SII levels were higher in the CSFP group. In addition, it has been reported that SII is superior to NLR and PLR in predicting the occurrence of certain cardiovascular diseases [10, 11, 30]. The present study also found no significant difference in PLR between the two groups and that NLR was not a predictor of CSFP in the regression model, suggesting that SII could better predict the occurrence of CSFP compared with NLR and PLR.

Diffuse atherosclerosis has also been shown to be an important causative factor in cardiovascular diseases such as CSFP. Avşar et al. [35] reported that carotid intima-media thickness (CIMT) was a marker of early atherosclerosis in blood vessels and that CIMT was significantly increased in patients with CSFP. It has also been demonstrated that NLR and SII are significantly associated with atherosclerosis, as in a study by Kaya et al. [36] who found NLR to be a predictor of severe coronary atherosclerosis and another study which showed that SII was one of the risk factors for atherosclerosis in predicting the severity of coronary artery lesions [10]. The present study also demonstrated that the NLR and SII levels were significantly higher in the CSFP group compared to the control group, and that SII was positively correlated with mTFC and increased with an increase in the number of coronary arteries involved. Therefore, SII may also assume an important role in the development of CSFP through atherosclerosis.

## Limitations

Our study has some potential limitations. Firstly, this study was a single-centre study with a relatively small sample size and no long-term follow-up of the patients with CSFP. Secondly, we did not measure C-reactive protein, an inflammatory mediator. Further confirmation is needed in more rigorous large-scale, prospective, and randomized controlled studies.

## Conclusions

In conclusion, the results of our study suggested that SII is an easily available and inexpensive new biomarker, and that higher levels of SII were significantly and independently related to the occurrence of CSFP. Moreover, its value increased as the number of involved vessels increased, Therefore, SII as a mediator of inflammation can predict the occurrence and severity of CSFP. we should pay attention to patients with high SII levels in clinical practice, and further studies are needed to confirm these results and the mechanism of action of SII in CSFP and further exploration is also needed in the treatment of CSFP.

## Future perspective

Coronary slow flow phenomenon (CSFP) is a common cardiovascular disease, but the pathophysiological mechanism is still unclear. Systemic immune-inflammation index (SII) as a new inflammatory biomarker in the present study can predict the occurrence of CSFP. Therefore, for patients with CSFP, SII would be important for the early diagnosis and stratification of the disease, also it would provide more insights into treatment in future clinical practice.

## Data Availability

The datasets generated and analysed during the current study are not publicly available due to the need for further research in this area but are available from the corresponding author on reasonable request.

## Abbreviations

SII: Systemic immune-inflammation index
CSFP: Coronary slow flow phenomenon
mTFC: The mean thrombolysis in myocardial infarction frame count
CAG: Coronary angiogram
CAD: Coronary artery disease
TFC: TIMI frame count
LAD: Left anterior descending artery
LCX: Left circumflex artery
RCA: Right coronary artery
NLR: Neutrophil to lymphocyte ratio
PLR: Platelet to lymphocyte ratio
ROC: Receiver operating characteristic
LDL: Low-density lipoprotein
HDL: High-density lipoprotein
NETs: Neutrophil extracellular traps
hs-CPR: High sensitive CRP
CIMT: Carotid intima-media thickness
TIMI: Thrombolysis in myocardial infarction
